# Umbilical cord-derived mesenchymal stem cell conditioned medium reverses neuronal oxidative injury by inhibition of TRPM2 activation and the JNK signaling pathway

**DOI:** 10.1007/s11033-022-07524-9

**Published:** 2022-05-18

**Authors:** Yan Wang, Jiaxin Liu, Baocong Yu, Yiran Jin, Jiahui Li, Xiaona Ma, Jianqiang Yu, Jianguo Niu, Xueyun Liang

**Affiliations:** 1 Key Laboratory of Ningxia Stem Cell and Regenerative Medicine, General Hospital of Ningxia Medical University, 750001 Yinchuan, China; 2 Ningxia Key Laboratory of Cerebrocranial Diseases, Ningxia Medical University, 750004 Yinchuan, China; 3 School of Pharmacology, Ningxia Medical University, 750004 Yinchuan, China

**Keywords:** Mesenchymal stem cells, Conditioned medium, TRPM2, Neuronal apoptosis, MAPK

## Abstract

**Background:**

The mechanism by which MSC-CM protects neuronal cells against ischemic injury remains to be elucidated. In this study, we aimed to clarify the protective effect of umbilical cord-derived mesenchymal stem cell conditioned medium (UC-MSC-CM) on neuronal oxidative injury and its potential mechanism.

**Methods and Results:**

Neuronal oxidative damage was mimicked by H2O2 treatment of the HT22 cell line. The numbers of cleaved-Caspase-3-positive cells and protein expression of Caspase-9 induced by H2O2 treatment were decreased by UC-MSC-CM treatment. Furthermore, SOD protein expression was increased in the MSC-CM group compared with that in the H2O2 group. The H2O2-induced TRPM2-like currents in HT22 cells were attenuated by MSC-CM treatment. In addition, H2O2 treatment downregulated the expression of p-JNK protein in HT22 cells, and this the downward trend was reversed by incubation with MSC-CM.

**Conclusions:**

UC-MSC-CM protects neurons against oxidative injury, possibly by inhibiting activation of TRPM2 and the JNK signaling pathway.

## Introduction

Stroke is the second leading cause of death worldwide and a major contributor to disability. Ischemic stroke, which is the most common type of this event[1], disrupts the production and clearance of reactive oxygen species (ROS), leading to their accumulation in brain tissue. This, in turn, results in the destruction or changes in lipids, proteins and nucleic acids in a cascade reaction that ultimately causes neuronal death[2]. However, no effective therapeutic strategies have yet been developed to prevent or treat oxidative stress injury.

Mesenchymal stem cells (MSCs) have been shown to exert antioxidant functions in experimental animals[3]. In recent years, MSCs have been widely used in experimental investigations of nerve injury repair because of their strong regeneration potential and ability to improve the microenvironment by secreting a variety of cytokines[4]. MSC-conditioned medium (MSC-CM) is heterogeneous and contains various soluble factors that can reduce lipid peroxidases generation, while promoting antioxidant enzyme production[5–8]. However, the mechanism by which MSC-CM protects neuronal cells against ischemic injury remains to be elucidated.

Transient receptor potential melatonin 2 (TRPM2) belongs to the TRP family and is a non-selective cation channel that allows the entry of calcium ions[9–11]. Studies have shown that H_2_O_2_ and ROS activate TRPM2 to induce the accumulation of calcium ions in cells, leading to cell death[12]. Importantly, recent studies have shown that TRPM2 channels play an important role in ischemic stroke, with TRPM2 activation further aggravating the condition[13–15].

In this study, we investigated the protective effect of umbilical cord-derived MSC-CM on H_2_O_2_-induced neuronal injury, and the potential roles of TRPM2 inhibition and the MAPK pathway in the underlying mechanism.

## Materials and methods

### HT22 cell culture

HT22 cells were purchased from Bena Chuang Lian Biotechnology Co., Ltd(Beijing, China).and cultured in Dulbecco’s Modified Eagle Medium (DMEM; Gibco, USA) containing 5% fetal bovine serum (FBS; Biological Industries, Israel) and incubated at 37℃ under 5% CO_2_ in a humidified atmosphere.

### Preparation of human umbilical cord-MSC-CM

This study was approved by the Ethics Committee of the General Hospital of Ningxia Medical University, China. Human umbilical cord-MSCs (UC-MSCs) were isolated from the umbilical cord connective tissue collected from healthy patients after obtaining informed consent. 3×10^5^ UM-MSCs were seeded in 100mm culture plate containing Ultra Culture Serum-free Medium (Lonza, Switzerland) supplemented with 2% Pall Ultroser G Serum Substitute (Pall, USA), and cultured in the CO_2_ incubator maintained at 37°C with 5% CO_2_ and a humidified atmosphere. Medium was changed every 3 days. At approximately 90% confluence, cells were passaged and 3×10^5^ UM-MSCs reseeded in a new 100mm culture plate. 1×10^6^ UC-MSCs at passages 4 were seeded in a new 100mm culture plate. At 60–70% confluence, the medium was changed and the cells cultured for a further 24 h before harvesting the conditioned medium (CM). The CM was filtered (0.22-µm pore size) to remove cellular debris and concentrated using ultrafiltration units with a 3-kDa molecular-weight cutoff (Millipore, Burlington, MA) to obtain the MSC-CM.

### Experimental design

HT22 cells were divided into the following experimental groups: Normal: culture in complete medium; H_2_O_2_: treated with 100 µM H_2_O_2_ for 1 h; MSC-CM: administration of UC-MSC-CM after treatment with 100 µM H_2_O_2_ for 1 h; SP600125 (a JNK inhibitor) and PD98059 (an ERK inhibitor): starved with 0.5% serum for 6 h, and cultured in the presence of the inhibitors for 24 h (SP600125 50 µM; PD98059 60 µM; MCE, USA); SP600125/PD98059 + H_2_O_2_: cultured in the presence of the inhibitors for 24 h before the addition of 100 µM H_2_O_2_ for 1 h; SP600125/PD98059 + MSC-CM: cultured in the presence of the inhibitors for 24 h before the addition of 100 µM H_2_O_2_ for 1 h, when UC-MSCs-CM were added.

### Enzyme-linked immunosorbent assay (ELISA)

Cystatin SA, CD200 R1, Pax3, HIF-1beta, Neuroligin2, FGF-21, HGF, VEGF, BDNF, EGF, GDNF and NT3 were detected in MSC-CM and Fibroblast-CM using commercial ELISA kits (Cloud-Clone Corp, China) according to the manufacturer’s instructions.

### Whole-cell patch clamp experiment

HT22 cells were cultured on Matrigel coated cover slides in 35mm culture dishes containing DMEM supplemented with 5% FBS. The prepared HT22 cell slides were recorded in the whole-cell mode at room temperature. The cells were perfused continuously with DMEM supplemented with 5% FBS. The pipette solution contained (mM): 125 potassium D-gluconate, 8 NaCl, 2 Mg ATP, 0.3 Na GTP, 0.2 EGTA and 10 HEPES, with the pH adjusted to 7.2–7.4 using KOH. The recording glass electrodes were pulled from the borosilicate glass using a P97 laser electrode puller (Sutter Instruments Company, USA), and the resistance between the electrode tip and cell membrane was 8–12 MΩ. Cells were held at a potential of − 60mV and current-voltage relations were obtained from voltage ramps from − 80 to + 80 mV (50 ms duration). Membrane currents were digitally sampled at a frequency of 10 kHz and low-pass filtered at 2–5 kHz. H_2_O_2_ (100 µM; Yantai, China) was used to activate the TRPM2 channels. Data acquisition was performed using EPC-10 Patch Clamp Amplifier (HEKA Elektronik, Germany). The results were analyzed and plotted using GraphPad Prism software, version 7.04.

### Immunofluorescence staining

HT22 cells were fixed for 10 min with 4% paraformaldehyde (Biotopped Life Sciences, China), washed with PBS and permeabilized with 0.5% Triton-X in PBS for 1 h at room temperature. Non-specific binding was reduced by incubation with blocking buffer (0.5% Triton-X in PBS + 5% normal goat serum) for 1 h at room temperature. Cells were then incubated overnight at 4°C with the following primary antibodies diluted in blocking buffer: anti-Caspase3 (1:1,000, cat. no. ab49822; Abcam, UK), anti-TRPM2 (1:500, cat. no. ab11168; Abcam). Cells were then incubated for 3 h at room temperature with the following fluorescently-labeled secondary antibody diluted in blocking buffer: Cy3-labeled goat anti-rabbit (1:1,000, cat. no 111-165-003; Jackson ImmunoResearch Laboratories, USA). Cells were incubated with 4′,6′ diamino-2-phenylindole dihydrochloride (DAPI) (1:4; Solarbio, China) for 3–5 min at room temperature and washed with PBS before collecting images under a fluorescence microscope (Olympus, Japan) in the dark. The positive cells were counted in 10 random microscopic fields of a 25mm culture dish from 3 independent experiments, averaged and expressed as % of total cells in a 200x field.

### Western blot analysis

Total proteins were extracted from the HT22 cells using IP Lysis buffer (cat. no 87787; Thermo, USA) with protease and phosphatase inhibitors (cat. no. 78441; Thermo, USA). The concentration was determined using the bicinchoninic acid (BCA) method (cat. no. P0012; Beyotime, China). Equal amounts of protein samples were separated by sodium dodecyl sulfate-polyacrylamide gel electrophoresis (SDS-PAGE) with 10% and 15% gels (cat. no. PG112 and PG114; Epizyme Biotechnology, China) and transferred to polyvinylidene fluoride membranes (Millipore, Burlington, MA, USA). The membranes were blocked with 5% non-fat milk for 2 h and incubated overnight at 4°C with the following primary antibodies: anti-SOD (1:1,000, cat. no. ab183881; Abcam), anti-pJNK1/2/3 (1:1,000, cat. no. ab219584; Abcam), anti-pERK1/2(1:1,000, cat. no. ab201015; Abcam) and anti-Caspase9 (1:1,000, cat. no. WL01551; Wanleibio, China), and anti-β-actin (1:10,000, cat. no. 66009-1-Ig; Proteintech, USA). The membranes were then incubated for 1 h at room temperature with the following secondary antibodies: IRDye680RD goat anti-rabbit (1:20,000, cat. no. 926-68071; Licor, USA), IRDye680RD goat anti-mouse (1:20,000, cat. no. 926-68180; Licor) and IRDye800CW goat anti-mouse (1:20,000, cat. no. 926-32351; Licor). The signal on the membrane were visualized by the Odyssey infrared scanner (Licor, USA) and optical densities were quantified by Image J software. β-actin was used as a loading control.

### Statistical analysis

Statistical analysis was performed using GraphPad Prism software, version 7.04. Data are presented as mean ± standard deviation (SD) of at least three experiments. One-way analysis of variance (ANOVA) and repeated measures ANOVA were used for multivariate data analyses. *P* < 0.05 was set as the threshold for statistical significance.

## Results

### UC-MSC-CM protects HT22 cells against H_2_O_2_-induced neuronal apoptosis

**Fig. 1:**
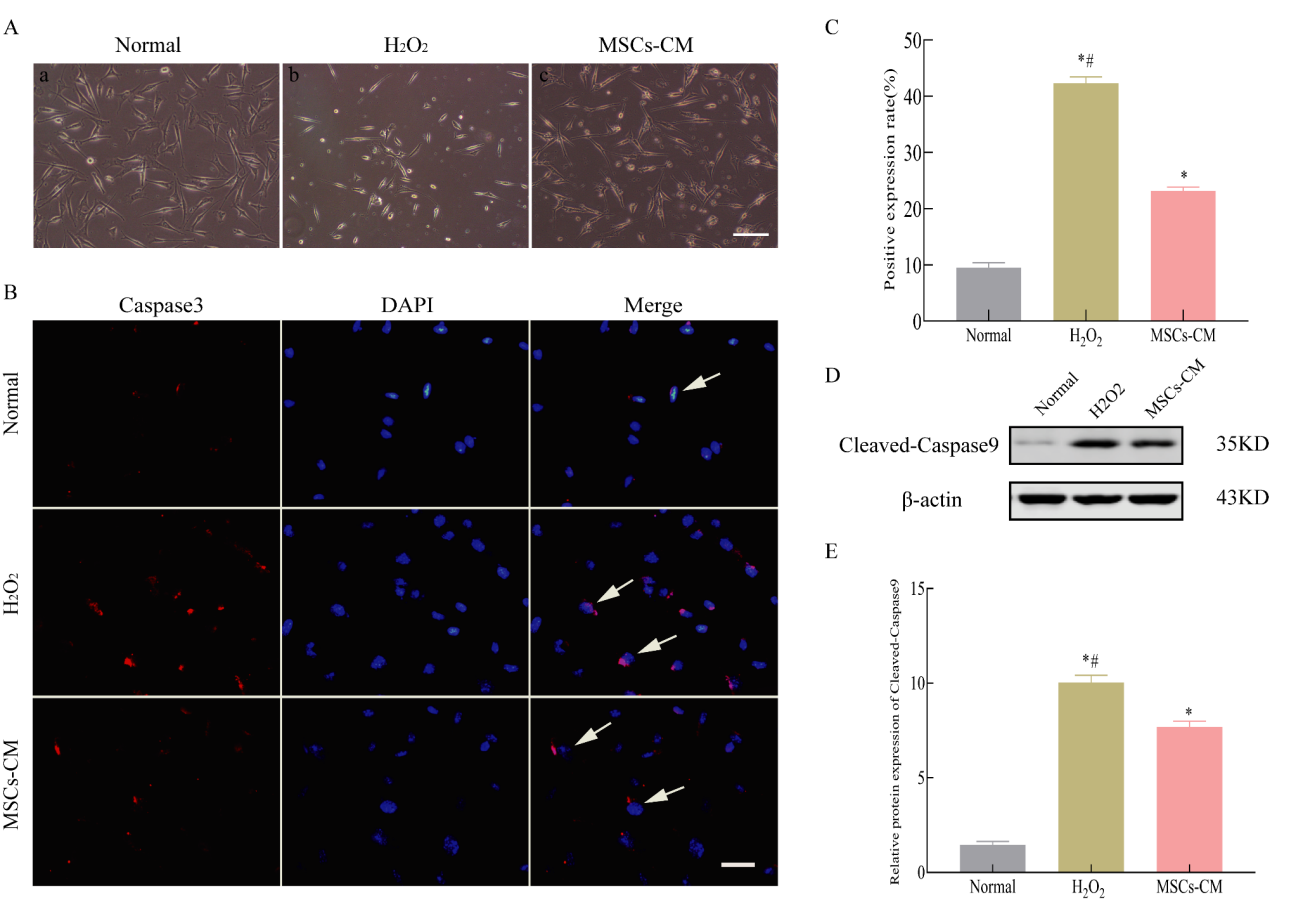
UC-MSCs-CM protected HT22 cells against H_2_O_2_-induced neuronal apoptosis. A: Representative images of HT22 cell morphology, scale bar = 100 μm; B: Representative images of immunofluorescence staining of cleaved Caspase-3, scale bar = 20 μm; C: Percentages of the Caspase-3-positive cells. Caspase-3 positive cells were counted in 10 random microscopic fields at 200× magnification of a 25 mm culture dish from 3 independent experiments, averaged and expressed as % of total cells in a field; D: Representative images of Western blot analysis of cleaved-Caspase-9; E: Relative expression analysis of cleaved-Caspase-9. Data represent the mean ± SD of 3 independent experiments. ^*^P < 0.05 compared with Normal group, ^#^*P* < 0.05 compared with MSCs-CM group

To examine the protective ability of UC-MSC-CM, we treated H_2_O_2_-stimulated HT22 cells with UC-MSC-CM for 24 h. Compared with the H_2_O_2_-treated group, the density of HT22 cells was higher after treatment with UC-MSC-CM, although there was no morphological difference between the cells in the two groups (*P* < 0.05, Fig. 1A). Fluorescence immunostaining showed that the number of Caspase-3 positive cells was significantly lower in the UC-MSCs-CM group compared with that in H_2_O_2_-treated group (*P* < 0.05, Fig. 1B and C). Furthermore, Western blot analysis showed that the expression level of cleaved-Caspase-9 protein was significantly lower in the UC-MSC-CM group compared with that in H_2_O_2_-treated group (*P* < 0.05, Fig. 1D and E).

### UC-MSC-CM is composed of cellular growth factors and mediates anti-oxidative functions

**Fig. 2:**
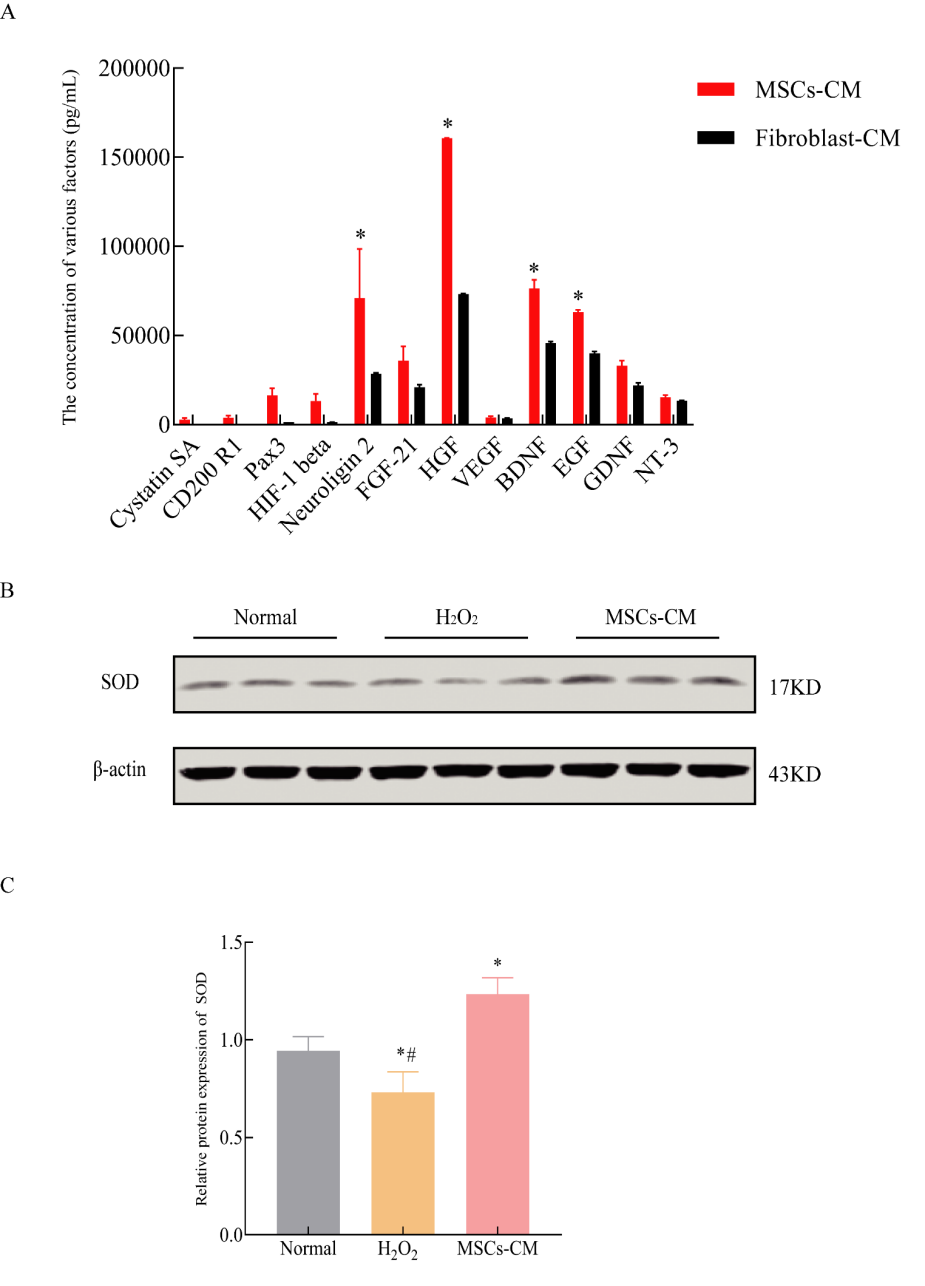
UC-MSC-CM is composed of cellular growth factors and possessed antioxidative ability. A: ELISA analysis of the concentration of various factors in the supernatant of MSCs-CM and fibroblast-CM; B: Representative images of Western blot analysis of SOD; C: Relative expression of SOD. Data represent the mean ± SD of 3 independent experiments. ^*^P < 0.05 compared with Normal group, ^#^*P* < 0.05 compared with MSCs-CM group

The cytotropic factors secreted into the UC-MSC-CM were analyzed by ELISA. Many growth factors, such as EGF, BDNF, GDNF and HGF, were detected in the UC-MSCs-CM (Fig. 2A). Western blot analysis showed that the expression level of SOD protein was significantly higher in UC-MSC-CM group compared with that in the H_2_O_2_-treated group (*P* < 0.05, Fig. 2B and C).

### UC-MSC-CM suppresses TRPM2-related currents

**Fig. 3:**
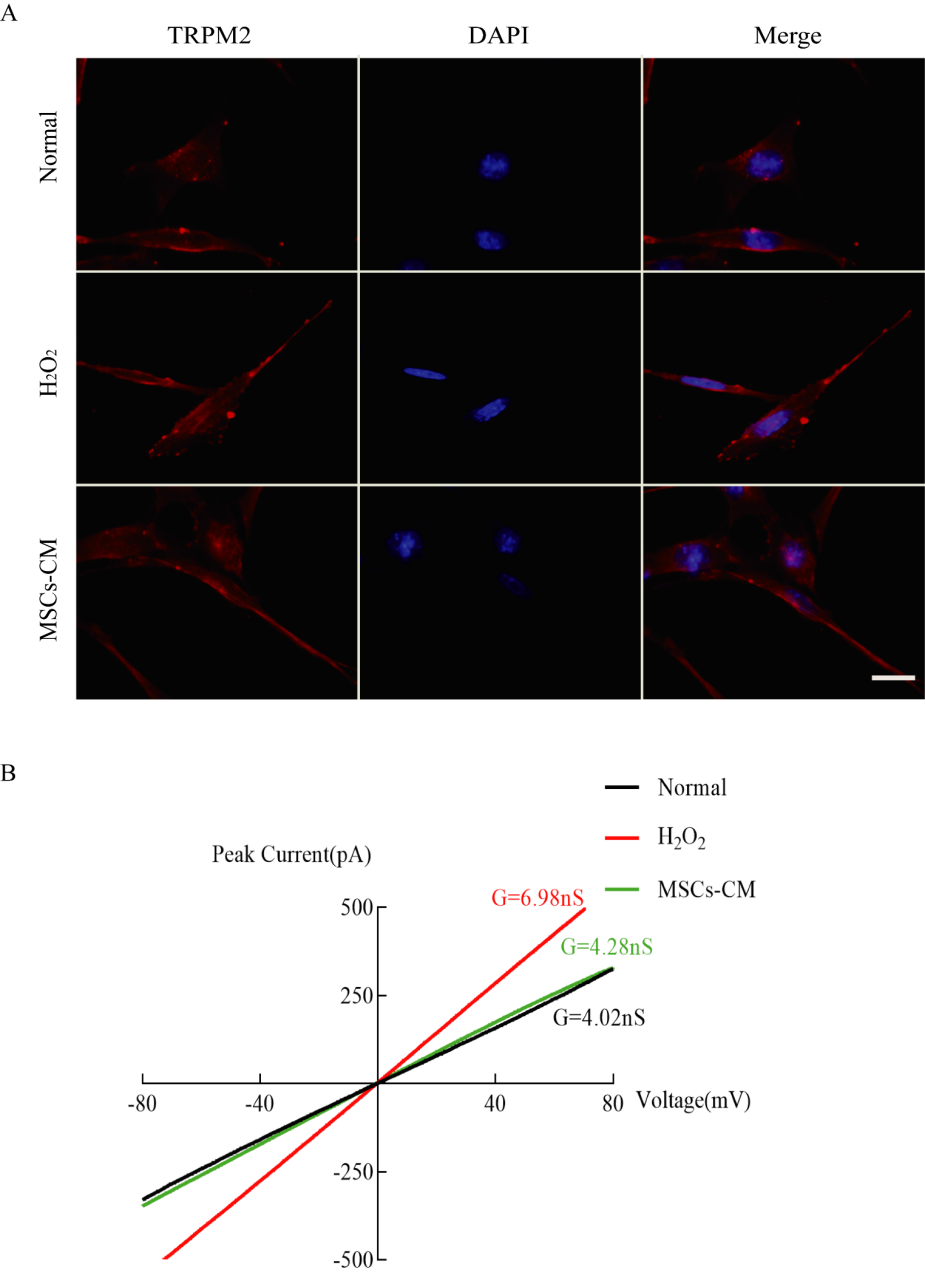
UC-MSC-CM suppressed TRPM2-related currents. A: Representative images of immunofluorescence staining of TRPM2, scale bar = 20 μm.; B: I-V curve of TRPM2 showing changes in membrane potential

TRPM2 can be activated by H_2_O_2_-induced oxidative stress[16]. Fluorescence immunostaining confirmed the expression of TRPM2 in HT22 cells, with no marked differences in the number of TRPM2-positive cells and the cellular location of TRPM2 between the groups (Fig. 3A). Whole-cell patch clamp measurement were showed that H_2_O_2_ stimulation of HT22 cells evoked a non-selective inward current with current–voltage properties similar to those that are characteristic of TRPM2[17]. This H_2_O_2_-induced TRPM2-like current in HT22 cells was attenuated by treatment with UC-MSC-CM (Fig. 3B)

### The neuroprotective effects of UC-MSC-CM are mediated via the JNK signaling pathway

**Fig. 4:**
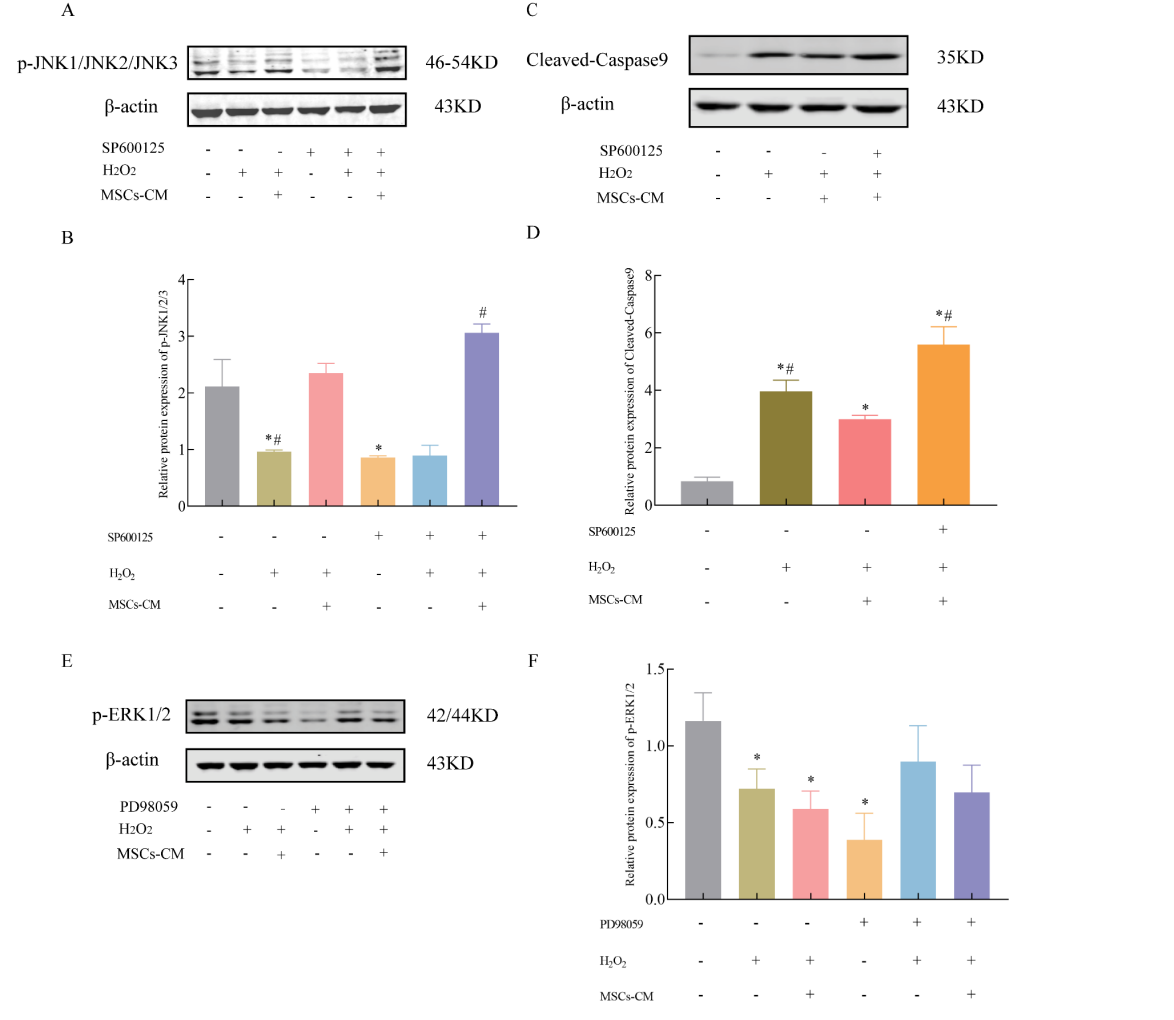
UC-MSCs-CM protection of HT22 cells was mediated via the JNK signaling pathway. A: Representative images of the Western blot analysis of the protein expression of p-JNK1/2/3 protein expression; B: Relative expression of p-JNK1/2/3; C: Representative images of the Western blot analysis of cleaved-Caspase-9 protein expression; D: Relative expression of cleaved-Caspase 9; E: Representative images of the Western blot analysis of p-ERK1/2 protein; F: Relative expression of p-ERK1/2. Data represent the mean ± SD of 3 independent experiments. ^*^P < 0.05 compared with Normal group

To explore the role of the H_2_O_2 -_related MAPKs signaling pathway in the mechanism by which UC*-*MSC-CM protects HT22 cells against H_2_O_2_-induced injury, we analyzed the expression of p-JNK, p-P38 and p-ERK proteins. Western blot analysis showed that the expression level of p-JNK protein was significantly decreased after treatment with H_2_O_2_ and that this trend was reversed by subsequent treatment with UC-MSC-CM (*P* < 0.05, Fig. 4A and B) accompanied by a decreased in the expression level of cleaved-Caspase 9 protein (*P* < 0.05, Fig. 4C and D). The expression level of p-ERK protein was also decreased after treatment with H_2_O_2_, and the levels were unchanged by subsequent treatment with UC-MSC-CM (Fig. 4D and E). The protein expression of p-P38 has not been detected in each group (data not shown).

## Discussion

Under conditions of cerebral ischemia, the excitotoxicity and subsequent ROS overproduction can damage neuronal structures, leading to cell death. Many studies have indicated that MSC transplantation is a promising therapy for cerebral ischemia. To date, several studies have evaluated the neuroprotective effects of MSC-CM in various diseases and many showed positive results[18]. Pre-clinically, MSC-CM has been shown to exert cellular protective and regenerative effects[19]. Recently, MSC-CM has been identified as one of the key components of the neuroprotective mechanisms of MSCs and can effectively ameliorate ischemia/reperfusion-induced brain injury by promoting angiogenesis, regulating immune responses, and inhibiting neuronal apoptosis [20, 21]. In accordance with other reports, we provided further evidence for MSC-CM treatment as a potential approach to the preservation of neuronal integrity and function. Our study showed that the MSC-CM treatment protected HT22 cells from H_2_O_2_-induced oxidative injury by decreasing apoptosis and increasing the cellular anti-oxidative ability.

Cerebral ischemia-induced TRPM2 activation triggers abnormal intracellular Ca^2+^ accumulation and cell death, which in turn causes irreversible brain damage[22]. Thus, TRPM2 has emerged as a new therapeutic target for ischemic stroke. TRPM2 inhibitors have been in preclinical development to prevent pathological Ca^2+^ overload[23–25]; however, successful development of specific inhibitors with good pharmacokinetic properties is a long and challenging process. Interestingly, we observed that MSC-CM contributes to the suppression of TRPM2 activation, indicating that MSCs secrete endogenous TRPM2 inhibitors. To identify such a component in the complex MSC-CM, we analyzed secreted protein expression and detected high levels many cytokines that may modulate the function of TRPM2 and warrant further investigation.

In eukaryotic cells, the MAPK signaling pathway regulates gene expression and processes such as cell proliferation, differentiation, apoptosis and stress response, and components of the pathway, including ERK, JNK, and p38, have been identified as therapeutic targets for many diseases[26–28]. Accumulating evidence supports a role for the MAPK signaling pathway in the pathogenesis and development of ischemic stroke[29, 30]. However, the upstream and downstream kinases of the MAPK signaling pathway are complex and have many influencing factors. Many studies have shown that the MAPK signaling pathway is the key modulator of ROS-related apoptosis[31, 32]. Therefore, we investigated the potential of MSC-CM to modulate the MAPK signaling pathway. Following treatment of HT22 cells with H_2_O_2_, expression levels of both p-ERK and p-JNK were decreased. Although the protein expression of p-P38 has not been detected in each group, we speculate that H_2_O_2_ may also induce the phosphorylated level of P38 decrease, but we could not observe the decreased p-P38 due to its low level in normal culture HT22 cells. These data indicated that H_2_O_2_ treatment could suppress the activation of MAPKs. Previous studies proved that ERK and JNK possess their upstream activators respectively[29, 30]. We showed that MSC-CM treatment effectively prevented the H_2_O_2_-induced decrease in p-JNK expression, but had no effect on p-ERK expression, our results suggested the component of MSC-CM might activate JNK, but not ERK. Furthermore, the protective effect of MSC-CM treatment against the H_2_O_2_-induced decrease in JNKs expression was accompanied by a decrease in neuronal cell apoptosis. These findings are consistent with previous reports that JNKs play roles in neuronal development and neurite growth[33, 34]. Interestingly, we have found SP600125 + MSC-CM group showed significant elevation of pJNK expression compared to MSC-CM group. Although SP600125 could block the activation of JNK, it also could increase the ROS level [35]. When the HT22 cells were treated with H_2_O_2_, a stronger oxidative stress was generated, cell physiology changes significantly once excessive amount of ROS accumulated. HT22 might become more susceptible to MSC-CM treatment, so the significant elevation of pJNK expression was observed. The detail mechanism still needs us to do further study.

In this study, we evaluated the effects of UC-MSCs-CM on neuronal oxidative damage. We showed that UC-MSCs-CM protects neurons against oxidative injury, possibly by inhibiting activation of the TRPM2 and JNK signaling pathway.

## Declarations

### Data accessibility statement

The data used to support the findings of this study are included in this article.
